# Evaluation of the Relationship between *5-HTT* and *MAO* Gene Polymorphisms, Mood and Level of Anxiety among Postmenopausal Women

**DOI:** 10.3390/ijerph120100268

**Published:** 2014-12-24

**Authors:** Elżbieta Grochans, Anna Jurczak, Małgorzata Szkup, Agnieszka Samochowiec, Anna Włoszczak-Szubzda, Beata Karakiewicz, Anna Grzywacz, Agnieszka Brodowska, Jerzy Samochowiec

**Affiliations:** 1 Department of Nursing, Pomeranian Medical University in Szczecin, 48 Żołnierska St., 71-210 Szczecin, Poland; E-Mails: jurczaka@op.pl (A.J.); m.szkup@onet.eu (M.S.); 2 Department of Clinical Psychology, Institute of Psychology, University of Szczecin, 69 Krakowska St., 71-017 Szczecin, Poland; E-Mail: samoaga@tlen.pl; 3 Department of Informatics and Health Statistics, Institute of Rural Health, 2 Jaczewskiego St., 20-090 Lublin, Poland; E-Mail: a-wlos@tlen.pl; 4 Department of Public Health, Pomeranian Medical University in Szczecin, 48 Żołnierska St., 71-210 Szczecin, Poland; E-Mail: karabea@sci.pum.edu.pl; 5 Department of Psychiatry, Pomeranian Medical University in Szczecin, 26 Broniewskiego St., 71-460 Szczecin, Poland; E-Mail: annagrzywacz@gazeta.pl; 6 Department of Gynaecology and Urogynaecology, Pomeranian Medical University in Szczecin, 2 Siedlecka St., 71-010 Police, Poland; E-Mail: agabrod@wp.pl

**Keywords:** mood disorders, postmenopause, *5HTTLPR*, MAO A, anxiety

## Abstract

*Objective*: The aim of this study was to analyze how mood and anxiety level are related to the functional genetic polymorphism in the promoter region of *SLC6A4* (*5-HTTLPR)* and the *30-bp VNTR* polymorphism in the *MAO A* promoter region. *Methods*: The study involved 272 postmenopausal women from Poland. The authors employed the State-Trait Anxiety Inventory for measuring levels of anxiety, the Mood Adjective Check List for measuring mood, and genetic tests. *Results*: Analysis did not show any statistically significant differences in the mean levels of anxiety, and mood disorders in women in relation to genotypes of the *5-HTTLPR (SLC6A4)* polymorphism and the *30-bp VNTR* polymorphism in the *MAO A* promoter region. However, these problems were more severe among women with *s/s* genotype. In the case of *MAO A* gene polymorphism, the level of anxiety was higher in women with a 4/4 genotype. *Conclusions*: The study did not prove the possibility of the identification of homogeneous groups of women with an elevated risk of developing anxiety and mood disorders during the post-menopausal period. Nevertheless, it showed that respondents with *s/s* genotype of the *44-bp* polymorphism in the *5-HTT (SLC6A4)* promoter region had the highest average anxiety levels both as a state and as a trait. Furthermore, the analysis of the *30-bp VNTR* polymorphism in the *MAO A* promoter region demonstrated slight differences in anxiety levels between the women, indicating that those with a 4/4 genotype had higher severity of anxiety symptoms.

## 1. Introduction

Menopause is the last menstruation in a woman’s life, usually occurring between the ages of 48–52. Health problems of postmenopausal women have become a tremendous challenge for both modern medicine and social sciences, because as much as 30% of the woman’s life falls within the postmenopausal period [1].

The most frequent climacteric symptoms are hot flushes and excessive sweating. Their occurrence is associated with changes in the levels of neurotransmitters, mainly adrenalin, serotonin, dopamine, and prostaglandins, acting locally at the central nervous system level [2].

Females are exposed to anxiety disorders including anxiety attacks (panic disorder) two to three times more frequently than males, especially during the perimenopausal period. The etiology of panic disorder covers the serotonergic, noradrenergic, γ-aminobutyric acid (GABA), and neuroanatomic models, as well as the genetic hypothesis (strong stressors are necessary).

Genes engaged in the serotonergic pathways are regarded as candidate genes due to the documented role of serotonin (*5-HT*) in the etiopathogenesis of mood disorders. One of the most often investigated genes of this group is the serotonin transporter gene (*SLC6A4*). A single gene (*SLC6A4*)*,* located on the human chromosome 17q11*.*2. 17q12, is coded by serotonin transporter (*5*-*HTT*). The polymorphism of this gene is characterized by the insertion or deletion of the *44-bp* sequence and this is related to the different transcriptional activity of the gene. Allele with *44-bp* deletion (short allele) is characterized by a three times lower transcriptional activity than allele with *44-bp* insertion (long allele) [3].

Available results indicate that the presence of this gene allele is associated with higher neuroticism as well as mood and anxiety disorders. When combined with adverse environmental factors, this increases the possibility of mood disorders as a reaction to stressful life events [4]. Carriers of the *s* allele of this gene show a greater susceptibility to affective disorders and states of anxiety [5].

The *MAO A* gene may also be responsible for an inclination to depression; this is an important enzyme associated with the metabolism of biogenic amines and neurotransmitters [6]. Monoamine Oxidase *A* (*MAO A*) plays a crucial role in the degradation of monoamines, such as serotonin and noradrenalin, which participate in the development of depression. It has also been confirmed that *MAO A* inhibitors, such as tranylcypromine or moclobemide, may be successfully applied in the treatment of depression [7]. Additionally, it was observed that bio-modulatory properties of serotonin re-uptake inhibitors may be translated into improvement of patient clinical outcomes beyond their anti-depressant action [8]. Sabol* et al.* were the first to describe the *uMAO A* polymorphism, which is the *VNTR* polymorphism, located in the promoter region of the *uMAO A* gene [7]. This consists of *30*-*bp* repeated sequences, which may be represented in 3, 3.5, 4 and 5 copies. Allele 3 is related to a lower transcriptional activity of the gene, while alleles 3.5 and 4 and 5 are associated with a higher *MAO A* activity [9]. The functionality of the repeat 5 allele is debated as functional characterization studies have demonstrated a significantly different enzyme activity, where alleles with 3.5 or 4 copies of the repeat sequence are transcribed more efficiently than alleles with 3R* in vitro*, while for the 5R allele controversial results have been reported [7,10].

The objective of this study is to analyze how the mood and the level of anxiety of postmenopausal women are related to the *5-HTTLPR (SLC6A4)* polymorphism and the *30-bp VNTR* polymorphism in the *MAO A* promoter region. The study presented here is the continuation and extension of the authors’ previous research into depression-related issues [11].

## 2. Material and Methods

### 2.1. Subjects

The study involved 272 healthy women from northern Poland who had their last menstrual period at least one year prior to the study. The criteria for inclusion in the study were at least one year since the last menstruation, normal smear test results, normal mammogram results, normal blood pressure, neither alcohol nor cigarette abuse, no current or past history of endocrinological disorders (such as diabetes or thyroid diseases), no current or past history of neoplastic diseases, no current or past history of psychiatric treatment.

The criteria for exclusion from the study were: abnormal smear test results, abnormal mammogram results, diagnosis of thyroid diseases and/or diabetes, diagnosis of neoplastic diseases, diagnosis of mental diseases.

Women with Axis I mental disorders (according to the ICD-10 classification) were excluded from the study by means of the PRIME-MD questionnaire and a psychiatric examination [12].

In 2011, a group of multi-discipline scientists from five countries assessed the cohort study conducted on middle-aged women, and accepted simplified criteria for the onset and the end of the menopausal transition period. The use of a STRAW +10 staging system is recommended regardless of age, race, BMI, and lifestyle. Patients included in the study were at the stage +2 STRAW +10,* i.e.*, late postmenopause. Stage +2 represents the period in which further changes in reproductive endocrine function are more limited and processes of somatic aging become of paramount concern [13,14].

All subjects gave their informed consent for inclusion in the research. The study was conducted in accordance with the Declaration of Helsinki, and the protocol was approved by the Bioethical Commission of the Pomeranian Medical University in Szczecin (permission number KB-0080/187/09).

### 2.2. Assessments

The first stage of the study was based on a diagnostic survey performed using standard research instruments:

State-Trait Anxiety Inventory (STAI) for measuring anxiety levels. This consists of two independent parts, each including a set of 20 questions. The first part, STAI (X-1), measures the level of anxiety understood as the transitory and situationally conditioned status of an individual. The second part, STAI (X-2), concerns anxiety understood as a relatively permanent personality trait [15]. Respondents take a stance on each statement, choosing one of four possible answers. The level of anxiety is expressed as the number of points obtained through summing up scores for separate answers. Scores for each part of the questionnaire may range from 20 to 80 points. Raw data are converted into standardized results for gender and age (stens). In this study, the authors used the 10-unit sten scale, interpreted as follows:

Scores of 1–4—low results (reflect a low level of anxiety as a trait and a state);
Scores of 5–6—average results (reflect a average level of anxiety as a trait and a state);
Scores of 7–10—high results (reflect a high level of anxiety as a trait and a state).

A high score for anxiety as a state may suggest stress associated with a difficult life s ituation, while a high score for anxiety as a trait indicates a permanent predisposition of a person to react with anxiety to life situations [15].

UWIST Mood Adjective Checklist (UMACL) for measuring mood understood as an affective experience of moderate duration, not related with the object or related with a quasi-object [16]. UMACL consists of a sheet with 29 printed adjectives. UMACL is divided into three sub-scales:

Hedonic tone—10 items;
Tense arousal—9 items;
Energetic arousal—10 items.

The raw result of every UMACL scale is the sum of points obtained for the items included in this scale. The answers given by the respondent are weighed on a four-point scale. In the hedonic tone scale the raw result ranges from 10 to 40 points, in the tense arousal scale—from 9 to 36 points, and in the energetic arousal scale—from 10 to 40 points. Higher scores suggest higher levels of particular mood. The scores are converted into stens: 1–4 stens—low results, 5–6—medium results, 7–10—high results.

If a high hedonic tone is accompanied by a low level of tense arousal and a moderately high level of energetic arousal, such a mood pattern is defined as a “positive mood”. The opposite pattern (a low level of a hedonic tone along with a high level of tense arousal and a low level of energetic arousal) is described as a “bad mood”. Both these patterns are characterized by matched mood dimensions. The third pattern is a configuration of a medium hedonic tone level, medium/low tense arousal, and medium energetic arousal—this pattern is described as an “average mood”. Other configurations of the levels of particular mood dimensions suggest unmatched mood dimensions [16].

The second stage of the study was based on genetic tests, in which DNA was isolated from whole blood by a salting-out method according to Miller [17]. A polymerase chain reaction (PCR) was used to identify DNA polymorphisms. The aim of the analysis was to amplify the fragment consisting of 2–5 repetitions of the *30-bp VNTR* polymorphism in the *MAO A* promoter region. The following primers were used F: 5’ CCC AGG CTG CTC CAG AAA 3’, R: 5’ GGA CCT GGG CAG TTG TGC 3’. The PCR consisted of an initial denaturing step at 95 °C for 3 min, followed by 34 cycles of denaturing at 94 °C for 40 s, annealing at 57 °C for 35 s, and polymerization at 72 °C for 50 s, with a final elongation step at 72 °C for 10 min.

The sizes of the amplified fragments were as follows: *239, 209, 226*, and *269-bp*. In the analysis of the *5 HTT* polymorphism, the fragment, including the *44-bp ins/del* in the regulatory sequence (the presence or the lack of *44-bp*), was amplified. The following primer sequences were used: *HTT F*, 5’ GGC GTT GCC GCT CTG AAT GC 3’; and *HTT R*, 5’ GAG GGA CTG AGC TGG ACA ACC AC 3’. The PCR consisted of an initial denaturing step at 94 °C for 5 min, followed by 30 cycles of denaturing at 94 °C for 55 s, annealing at 55 °C for 50 s, and polymerization at 72 °C for 60 s, with a final elongation step at 72 °C for 10 min. The sizes of the amplified fragments were *484* and *528-bp*. The PCR products were electrophoresed on 3% agarose gel, which was followed by ethidium bromide staining to detect the alleles [18,19].

### 2.3. Statistical Analysis

Statistical analysis was performed using STATISTICA 7.1 PL (StatSoft, Poland). The chi-squared independence test was applied to verify the null hypothesis regarding the independence of the variables analyzed. The authors also used one-factor analysis of variance (ANOVA) to verify the hypothesis—more than two groups of women are identical in terms of a specific quantitative variable. The results of the analysis were presented as F statistics and testing probability. The accepted significance level was α = 0.05. The power calculated for all the genetic tests exceeded 0.95 (*p* > 0.95).

## 3. Results

The authors of this study investigated two groups of potential contributors to the incidence of depressive symptoms in postmenopausal women. The functional genetic polymorphism in the promoter region of *SLC6A4* (*5-HTTLPR)* and the *30-bp VNTR* polymorphism in the *MAO A* promoter region:

With reference to the incidence of anxiety as a state and as a trait according to STAI X-1 and X-2;

With reference to the severity of mood changes according to UMACL.

The average age of the women in the study was 55.4 ± 3.5 years. More than a half (53.7%) had completed secondary education, 36%—higher education, 9.2%—vocational education, and 1.1%—primary education. Most of the women lived in urban areas with a population of more than 100,000 residents (60.3%); 9.2% and 7.3% lived in rural areas and towns of up to 10,000 residents, respectively; the remainder (23.2%) lived in towns with less than 100,000 residents. The vast majority of the participants in the study had life partners (73.2%).

While evaluating anxiety as a state and anxiety as a trait, it was found that women who showed these traits constituted the smallest group: 26.1% and 23.2%, respectively. The majority of respondents had either low or medium level of anxiety as a state—39.0% and 34.9%, and anxiety as a trait—42.6% and 34.2%, respectively. The evaluation of the respondents’ mood according to UMACL confirmed a lack of mood adjustment in nearly half of the women in the study (46.7%). In the remaining subgroups, a positive mood prevailed (31.2%); lowered mood was observed in 11.4% and an average mood in 10.7%.

The analysis of the data did not show any statistically significant differences in the mean levels of anxiety as a state and as a trait assessed with STAI in relation to genotypes and alleles of the *5-HTTLPR (SLC6A4)* polymorphism and the *30-bp VNTR* polymorphism in the *MAO A* promoter region. However, the highest mean values, compared to the reminder, were noted in women with *s/s* genotype, both in the evaluation of anxiety as a state (38.73 ± 11.81) and as a trait (46.06 ± 10.16). Furthermore, in the case of *MAO A* gene polymorphism, slight differences were observed, which evidenced a higher intensity of anxiety in women with a 4/4 genotype, in the assessment of anxiety as a state (38.43 ± 10.81) (Table 1).

**Table 1:** Basic descriptive statistics concerning anxiety as a state according to STAI X-1 and anxiety as a trait, according to STAI X-2 of the 44-bp polymorphism in the *5-HTTLPR (SLC6A4)* promoter region and of the *30-bp VNTR* polymorphism in the* MAO A* promoter region among women in the study.

Genotypes	Anxiety as a State				Anxiety as a Trait			
	n	χ ± SD	F	*p*	n	χ ± SD	F	*p*
	*5-HTT*				*5-HTT*			
***s/s***	33	38.73 ± 11.81	0.62	n.s.	33	46.06 ± 10.16	0.083	n.s.
***l/s***	108	38.58 ± 10.83			108	45.60 ± 9.73		
***l/l***	129	37.16 ± 9.71			129	45.35 ± 8.42		
**Total**	270	38.01 ± 10.52			270	45.53 ± 9.15		
	***MAO A***				***MAO A***			
**4/4**	119	38.43 ± 10.81	0.21	n.s.	119	44.36 ± 7.41	1.149	n.s.
**3/4**	117	37.84 ± 10.31			117	44.92 ± 8.77		
**3/3**	36	37.22 ± 10.27			36	45.48 ± 8.30		
**Total**	272	38.01 ± 10.50			272	45.51 ± 9.14		

n—number of respondents in genotype subgroup; χ ± SD—mean ± standard deviation; F—analysis of variance (ANOVA); *p*—level of significance determined for F; n.s.— non-significant.

Among the women examined, no statistically significant differences were noted in the distribution of genotypes and alleles of the *5-HTTLPR (SLC6A4)* polymorphism and the *30-bp VNTR* polymorphism in the *MAO A* promoter region while evaluating anxiety as a state and as a trait. Nevertheless, it was found that the *s/s* genotype and s allele occurred more frequently in women in whom the presence of anxiety as a state and as a trait was confirmed (Table 2). The 4/4 genotype and allele 4 was more common in women with intensified anxiety as a state. Similarly, but also statistically insignificantly, this was further observed in women with anxiety as a trait (Table 3).

**Table 2:** Distribution of genotypes and alleles frequency of the polymorphism in the *5-HTTLPR (SLC6A4)* promoter region, and intensity of anxiety as a state acc. to STAI X-1 and as a trait acc. to STAI X-2.

Anxiety Level	n	Genotypes			Alleles	
		*s/s* n (%)	*l/s* n (%)	*l/l* n (%)	*l* n (%)	*s* n (%)
**Anxiety as a State according to STAI X-1**						
**Low**	105	12 (11.4)	40 (38.1)	53 (50.5)	146 (69.5)	64 (30.4)
**Average**	94	9 (9.6)	41 (43.6)	44 (46.8)	129 (68.6)	59 (31.4)
**High**	71	12 (16.9)	27 (38.0)	32 (45.1)	91 (64.1)	51 (35.9)
		χ^2^ = 2.62, *p* (n.s.)			χ^2^ = 1.24, *p* (n.s.)	
**Anxiety as a Trait according to STAI X-2**						
**Low**	115	12 (10.4)	49 (42.6)	54 (47.0)	157 (68.3)	73 (31.7)
**Average**	92	12 (13.0)	33 (35.9)	47 (51.1)	127 (69.0)	57 (31.0)
**High**	63	9 (14.3)	26 (41.3)	28 (44.4)	82 (65.1)	44 (34.9)
		χ^2^ = 1.56, *p* (n.s.)			χ^2^ = 0.58, *p* (n.s.)	

n—number of respondents in genotype subgroup; χ^2^—Pearson Chi-square test; *p*—level of significance determined for χ^2^; n.s.—non-significant.

**Table 3:** Distribution of genotypes and alleles frequency of the *30-bp VNTR* polymorphism in the *MAO A* promoter region, and the severity of anxiety as a state according to STAI X-1 and as a trait acc. to STAI X-2.

Anxiety Level	n	Genotypes			Alleles	
		4/4 n (%)	3/4 n (%)	3/3 n (%)	4 n (%)	3 n (%)
**Anxiety as a State according to STAI X-1**						
**Low**	106	49 (46.2)	44 (41.5)	13 (12.3)	142 (67.0)	70 (33.0)
**Average**	95	36 (37.9)	43 (45.3)	16 (16.8)	115 (60.5)	75 (39.5)
**High**	71	34 (47.9)	30 (42.2)	7 (9.9)	98 (69.0)	44 (31.0)
		χ^2^ = 2.97, *p* (n.s.)			χ^2^ = 3.04, *p* (n.s.)	
**Anxiety as a Trait according to STAI X-2**						
**Low**	116	50 (43.1)	53 (45.7)	13 (11.2)	153 (66.0)	79 (34.0)
**Average**	93	38 (40.9)	36 (38.7)	19 (20.4)	112 (60.2)	74 (39.8)
**High**	63	31 (49.3)	28 (44.4)	4 (6.3)	90 (71.4)	36 (28.6)
		χ^2^ = 7.5, *p* (n.s.)			χ^2^ = 4.25, *p* (n.s.)	

n—number of respondents in genotype subgroup; χ^2^—Pearson Chi-square test; *p*—level of significance determined for χ^2^; n.s.—non-significant.

No statistically significant relationship was found between the qualitative assessment of mood among postmenopausal women according to UMACL, and the distribution of genotypes and allele frequency of the *5-HTTLPR (SLC6A4)* polymorphism *p* (n.s.). The *s/s* genotype was considerably less common in women in a good mood and in the case of a lack of mood dimension adjustment, compared with the remaining genotypes (*l/l* and *l/s*). Furthermore, among women in a poor mood, the percentage of *s/s* genotype did not significantly differ from the remainder, and was considerably higher (20.0%) than the percentage of this genotype in the groups of women in a better mood (from 10.3 %–13.3%). No statistically significant relationship was confirmed between the qualitative mood assessment according to UMACL and the distribution of genotypes and alleles of the *30-bp VNTR* polymorphism in the *MAO A* promoter region *p* (n.s.). In women in an average mood, allele 3 occurred considerably more frequently than in the remaining mood groups (48.3% and from 31.9%–35.5%, respectively) (Table 4).

**Table 4:** Distribution of genotypes and alleles frequency of the *5-HTTLPR (SLC6A4)* polymorphism and the *30-bp VNTR* polymorphism in the *MAO A* promoter region and mood state according to UMACL among the women examined.

Mood according to UMACL	n	*s/s* n (%)	*5-HTT*		*5-HTT*	
			Genotypes		Alleles	
			*l/s* n (%)	*l/l* n (%)	*l* n (%)	*s* n (%)
**Positive mood**	85	13 (15.3)	34 (40.0)	38 (44.7)	109 (64.9)	59 (35.1)
**Bad mood**	30	6 (20.0)	10 (33.3)	14 (46.7)	38 (63.3)	22 (36.7)
**Lack of adjustment of mood dimensions**	126	13 (10.3)	53 (42.1)	60 (47.6)	173 (68.7)	79 (31.3)
			χ^2^ = 2.69, *p* (n.s.)		χ^2^ = 0.99, *p* (n.s.)	
**Average mood**	29	1	11	17	45	13
	**n**	**4/4** **n (%)**	***MAO A***		***MAO A***	
			**Genotypes**		**Alleles**	
			**4/3** **n (%)**	**3/3** **n (%)**	**4** **n (%)**	**3** **n (%)**
**Positive mood**	85	39 (45.9)	34 (40.0)	12 (14.1)	112 (65.9)	58 (34.1)
**Bad mood**	31	13 (42.0)	14 (45.1)	4 (12.9)	40 (64.5)	22 (35.5)
**Average mood**	29	8 (27.6)	14 (48.3)	7 (24.1)	30 (51.7)	28 (48.3)
**Lack of adjustment of mood dimensions**	127	59 (46.5)	55 (43.3)	13 (10.2)	173 (68.1)	81 (31.9)
		χ^2^ = 5.98, *p* (n.s.)			χ^2^ = 5.64, *p* (n.s.)	

n—number of respondents in genotype subgroup; χ^2^—Pearson Chi-square test; *p*—level of significance determined for χ^2^.

## 4. Discussion

In the menopausal transition period most women experience symptoms of distress, such as: vasomotor complaints, poor sleep, vaginal dryness, depressed mood [20], feelings of irritability, anxiety, and decreased interest in sexual activity, which make a considerable contribution to the lowering of their quality of life [21].

Mood disorders and depressive symptoms are especially common among menopausal women. In their study, Freeman* et al.* demonstrated that a diagnosis of depressive disorder was 2.5 times more likely to occur in women in the menopausal transition period than in their premenopausal counterparts [22]. This also results in an increase in the number of hospitalizations due to major depressive disorders [23]. Frey* et al.* analyzed the contribution of cerebral mechanisms or functional changes to an increased risk of depression and anxiety in perimenopausal women. Their study, based on functional magnetic resonance imaging suggests that changes in brain networking and emotional regulation during the peri- and postmenopausal periods may be a part of an underlying mechanism, in the occurrence of mood and anxiety symptoms [24].

Many authors emphasize the relationship between a decline in progesterone and estrogen levels and the occurrence of mood disorders [21,22,24,25], as well as changes in emotional reactivity to stressful life events [26]. Ovarian and exogenous sex steroid hormones are likely to influence mood and cognition through the modulation of the system of neurotransmitters (e.g., serotonin, *5-HT*) [25]. Genetic variation of the serotonin transporter (*5-HTT)* gene is regarded as a factor which is probably associated with susceptibility to depressive disorders. The promoter region of the *5-HTT* gene (*SLC6A4*) has a functional polymorphism (*5-HTTLPR*), consisting of an insertion/deletion of a *44-bp* sequence. The presence of short allele results in a lower transcriptional activity of the *5-HTT* gene, and—consequently—individual differences in sensitivity to daily life stress. This relationship has been observed both in human and animal studies [27]. The role of serotonin in the etiopathogenesis of mood disorders has been documented, and the *SLC6A4* gene encoding for the serotonin transporter *(5-HTT)* is among the most frequently investigated genes in depressive symptoms [28].

The results of the study described in this article did not demonstrate any influence of the *5-HTTLPR (SLC6A4)* polymorphism on the mood and the level of anxiety of menopausal women. Findings reported by other researchers indicate a high relationship between the presence of *s/s* genotype or s allele, and a greater susceptibility to affective disorders, and, when combined with adverse environmental effects, also an increased probability of the development of depression [4].

The research conducted on a group of 102 mentally healthy women showed that those with *s* allele had significantly higher scores both on scales measuring depression (Zung Self-rating Depression Scale, ZSDS) and anxiety (Spielberger State-Trait Anxiety Inventory, STAI). Patients with *s/s* and *s/l* genotypes had significantly higher levels of depression, compared with respondents with the *1/1* genotype. The level of anxiety was significantly higher in women with s/l allele than those with the *1/1* genotype. The study presented here indicates that even in the group of mentally healthy individuals a relationship was noted between the *5-HTTLPR* gene polymorphism and inclination towards depression and anxiety [29].

The *5-HTTLPR* gene polymorphism also exerts an effect on the development of anxiety disorders as a result of the occurrence of strong stressors in childhood [15]. A similar relationship was observed with respect to the inclination to begin the consumption of alcohol at an early age [30].

This study conducted among healthy women at postmenopausal age did not confirm the above-mentioned results. However, an insignificant but frequent occurrence of the *s/s* genotype was observed in the group of respondents with a diagnosis of anxiety as a state and as a trait. Furthermore, the mean score values in the evaluation of anxiety, despite being statistically insignificant, were the highest among women possessing the *s/s* genotype.

A study conducted on a group of 350 students who were asked to register stress factors in daily life proved the presence of a relationship between the *5-HTTLPR* genotypes and anxiety on stressful days. Individuals with *l/l* genotype responded to stress factors less emotionally than carriers of the *l/s* or *s/s* genotypes [17]. Many studies confirmed the presence of a relationship between the *5-HTTLPR* polymorphism and an increased risk of the development of depression or symptoms of mood disorders in relation to stressful life events [31]. It has been proved in a study conducted on a group of 630 postmenopausal women by Grochans* et al.* that s allele of the *44-bp 5-HTTLPR (SLC6A4)* polymorphism was significantly more common in women with severe climacteric symptoms. Furthermore, it was observed that more severe climacteric symptoms typical of the menopausal period are significantly more common in women with s allele of the *44-bp VNTR* polymorphism in the *5-HTT (SLC6A4)* promoter region [11].

Despite these results, the study presented here did not confirm a higher severity of mood disorders in relation to stressful situations; this is the peri- and postmenopausal period in which various stress-inducing situations are common.

The study presented also covered an evaluation of the relationship between the frequency of occurrence of genotypes and alleles of the *30-bp VNTR* polymorphism in the *MAO A* promoter region, and the intensification of anxiety and mood among postmenopausal women. According to the literature, monoamino oxidase A* (MAO A)* plays a crucial role in the degradation of monoamines, such as serotonin and noradrenalin, which participate in the development of depression [32]. Rekkas* et al.* noticed a change in a central biomarker *(MAO A)* during perimenopausal age, which was comparable with changes occurring during major depressive episodes, and indicates a high-risk of major depressive episodes. The functions of *MAO A* influence oxidative stress and apoptosis. Those processes are regarded as excessive in both mood disorders and dementia [33]. Interesting results were obtained in studies which confirmed a U-shaped relationship between the activity of *MAO A* in blood platelets and behavior. It was found that both in individuals with low and those with high *MAO A* activity a greater impulsiveness may be observed in neuropsychological tests. In these individuals, the probability of addiction to tobacco and the development of anxiety disorders was significantly higher [34]. The analysis of the relationship between the severity of climacteric and depressive symptoms and the *30-bp VNTR* polymorphism in the *MAO A* promoter region demonstrated that women with allele “4” were at the highest risk of developing depressive symptoms [11]. Some interesting conclusions were drawn by Nilsson* et al.*, who carried out a large-scale study on 17–18-year-old students. They observed that the *MAO A u-VNTR* combined with psychosocial risk factors, was related to high alcohol consumption. However, these relationships differed between genders. A higher risk of being a high alcohol consumer was noted in female carriers of the long *MAO A u-VNTR* variant, whereas in boys, the risk was higher when they had the s allele. This study proved that there is the relationship between *MAO A u-VNTR* and gender in the gene-environment interaction [18].

The conclusions drawn from the analyses concerning the relationship between the *MAO A* gene polymorphism, and a tendency for affective disorders to develop, are not obvious. Some researchers hold the opinion that the presence of high activity *MAO A* polymorphism increases the risk of the development of depression [35]. A similar effect of this genotype on the frequency of undertaking suicidal attempts was also observed [36]. However, this study did not confirm a significant relationship between the frequency of genotypes and alleles of the *30-bp VNTR* polymorphism in the *MAO A* promoter region, and mood and higher levels of anxiety as a state and as a trait.

### Limitations

In this study the assessment of the mood and the level of anxiety of postmenopausal women was performed only once. The analysis did not demonstrate a clear relationship between mood and anxiety level and the presence of the *5-HTTLPR (SLC6A4)* polymorphism and the *30-bp VNTR* polymorphism in the *MAO A* promoter region. It could be useful to repeat such research in the same group over several time periods, in order to observe changes in mood and levels of anxiety over time. Therefore, the authors intend to continue the research one and five years from the first study.

*5-HTT rs25531*, crucially moderating the effect of *5-HTTLPR*, was not analyzed along with *5-HTTLPR*. No correction for multiple testing was applied. Our findings did not demonstrate a relationship between genetic factors and mood and anxiety level. It is worth emphasizing that participants of this study were selected very carefully and only healthy ones were included. Understanding of these issues could be extended by research on selected women with a clinical diagnosis of depression.

Despite all its limitations, which do not allow us to draw any conclusions for the general population, this is a relatively innovative study presenting a new and unexplored research area. Further research is necessary to confirm or reject the proposed hypothesis.

## 5. Conclusions

The study did not prove the possibility of the identification of homogeneous groups of women with an elevated risk of developing anxiety and mood disorders during the post-menopausal period. Nevertheless, it showed that respondents with the *s/s* genotype of the *44-bp* polymorphism in the *5-HTT (SLC6A4)* promoter region had the highest average anxiety levels both as a state and as a trait. Furthermore, the analysis of the *30-bp VNTR* polymorphism in the *MAO A* promoter region demonstrated slight differences in anxiety levels between the women, indicating that those with a 4/4 genotype had higher severity of anxiety symptoms.
